# DNA barcoding for bio-surveillance of emerging pests and species identification in Afrotropical Prioninae (Coleoptera, Cerambycidae)

**DOI:** 10.3897/BDJ.9.e64499

**Published:** 2021-04-28

**Authors:** Marion Javal, John S Terblanche, Desmond E Conlong, Norbert Delahaye, Elizabeth Grobbelaar, Laure Benoit, Carlos Lopez-Vaamonde, Julien M Haran

**Affiliations:** 1 Centre for Invasion Biology, Department of Conservation Ecology & Entomology, Faculty of AgriSciences, Stellenbosch University, Stellenbosch, South Africa Centre for Invasion Biology, Department of Conservation Ecology & Entomology, Faculty of AgriSciences, Stellenbosch University Stellenbosch South Africa; 2 South African Sugarcane Research Institute, Mount Edgecombe, South Africa South African Sugarcane Research Institute Mount Edgecombe South Africa; 3 Unaffiliated, Plaisir, France Unaffiliated Plaisir France; 4 Biosystematics Division, ARC-Plant Protection Research Institute, Private Bag X134, Queenswood, Pretoria, South Africa Biosystematics Division, ARC-Plant Protection Research Institute, Private Bag X134, Queenswood Pretoria South Africa; 5 CBGP, Cirad, Montpellier SupAgro, INRA, IRD, Univ. Montpellier, Montpellier, France CBGP, Cirad, Montpellier SupAgro, INRA, IRD, Univ. Montpellier Montpellier France; 6 INRAE, URZF, Orleans, France INRAE, URZF Orleans France; 7 IRBI, UMR 7261, CNRS-Université de Tours, Tours, France IRBI, UMR 7261, CNRS-Université de Tours Tours France

**Keywords:** Africa, Barcode Index Number (BIN), beetles, biodiversity, BOLD, biomonitoring, *Cacosceles newmannii*, Gabon, invasion biology, Madagascar, Republic of the Congo, South Africa, sugarcane.

## Abstract

DNA barcoding has been succesfully used for bio-surveillance of forest and agricultural pests in temperate areas, but has few applications in the tropics and particulary in Africa. *Cacosceles newmannii* (Coleoptera: Cerambycidae) is a Prioninae species that is locally causing extensive damage in commercially-grown sugarcane in the KwaZulu-Natal Province in South Africa. Due to the risk of spread of this species to the rest of southern Africa and to other sugarcane growing regions, clear and easy identification of this pest is critical for monitoring and for phytosanitary services. The genus *Cacosceles* Newman, 1838 includes four species, most being very similar in morphology. The damaging stage of the species is the larva, which is inherently difficult to distinguish morphologically from other Cerambycidae species. A tool for rapid and reliable identification of this species was needed by plant protection and quarantine agencies to monitor its potential abundance and spread. Here, we provide newly-generated barcodes for *C. newmannii* that can be used to reliably identify any life stage, even by non-trained taxonomists. In addition, we compiled a curated DNA barcoding reference library for 70 specimens of 20 named species of Afrotropical Prioninae to evaluate DNA barcoding as a valid tool to identify them. We also assessed the level of deeply conspecific mitochondrial lineages. Sequences were assigned to 42 different Barcode Index Numbers (BINs), 28 of which were new to BOLD. Out of the 20 named species barcoded, 11 (52.4%) had their own unique Barcode Index Number (BIN). Eight species (38.1%) showed multiple BINs with no morphological differentiation. Amongst them, *C. newmannii* showed two highly divergent genetic clusters which co-occur sympatrically, but further investigation is required to test whether they could represent new cryptic species.

## Introduction

There has been an increase in newly-emerged insect pests in recent years (Roques 2010) that can have a negative ecological and economic impact (Colautti et al. 2006, Kenis et al. 2009, Pimentel et al. 2001, Pimentel et al. 2005). There is an urgent need for the development of advanced tools for the early detection and accurate identification of new or emerging insect pests. One major problem is that many interceptions of newly-emerging insect pests are immature stages that are difficult or impossible to rapidly identify to species level (Wu et al. 2017). DNA barcoding is a tool for species identification, based on the use of a fragment of the cytochrome C oxidase 1 (COI) gene (Hebert et al. 2003). The divergence rate of DNA barcodes makes it possible to discriminate species for the vast majority of insects, which provides effective support for identification of individuals at the species level (Hebert et al. 2003). Although it has been criticised for low precision in certain cases (Moritz and Cicero 2004, Meyer and Paulay 2005, Song et al. 2008), its broad use makes it possible to assign an individual to a species regardless of its phenotype or the developmental stage or the state of the specimen collected. It effectively supplements taxonomic studies, based only on morphological criteria (Hajibabaei et al. 2007), provided that the data deposited in libraries are reliable and well curated (Kelnarova et al. 2018). DNA barcoding is, therefore, often used as a supporting tool to identify species or stages which are morphologically difficult to distinguish, especially in a context of invertebrate pest management. It can contribute to more rapid identification of insect pests (Ball and Armstrong 2008, Hodgetts et al. 2016, Wu et al. 2017) and has successfully been used as a tool for their bio-monitoring (Frewin 2013, Ashfaq and Hebert 2016). The online data base Barcode of Life Data Systems (BOLD, www.boldsystems.org) allows the automatic identification of species, based on their DNA barcodes (Ratnasingham and Hebert 2007, Ratnasingham and Hebert 2013). The development of the Barcode Index Number System (BINs) in BOLD further allows the automatic assignment of barcode sequences to genetic clusters, generating a web page for each cluster. Since clusters show high concordance with species (Lopez-Vaamonde et al. 2021), this system can be used as a proxy for species when taxonomic information is missing.

Cerambycidae are forest insects that play a major role in the decomposition of dead wood. Some species in this family also cause damage to a wide range of economically-important tree species (Haack et al. 2010, Eyre and Haack 2017) and can be economic pests of commercially-grown crops (Wang 2017). Cerambycids are uncommon pests of sugarcane crops (Carnegie and Conlong 1994), but some species have been found to cause severe damage in fields worldwide (Oyafuso et al. 2002,Mukunthan and Nirmala 2002, Dolinski et al. 2006). For example, in Thailand, the cerambycid *Dorysthenes buqueti* (Guérin-Méneville, 1844) showed a ten-fold population increase in sugarcane crops within a year causing significant damage (Pliansinchai et al. 2007). The Prioninae species *Cacosceles newmannii* (Thomson, 1877) is another recent example of a sugarcane pest. This species is native to Mozambique, Eswatini and South Africa and its biology has been poorly studied (Ferreira 1980; but see recent focused research efforts in, for example, Javal et al. 2018, Javal et al. 2019a, Javal et al. 2019b, Smit et al. 2021a, Smit et al. 2021b, Lehmann et al. 2021). The larval stage is thought to last for two years, during which larvae actively feed on root material and stem tissue (Way et al. 2017). Adults, on the other hand, have a very short life span of a few months and do not feed (Javal & Conlong, personal observations). Its host range has not yet been fully determined, even though preliminary studies show that the species is likely to be polyphagous (Smit et al. 2021b). Unlike many other Prioninae species, however, *C. newmannii* larvae are able to feed on living tissue and were found for the first time in 2015 in commercially-grown sugarcane in the KwaZulu-Natal (KZN) Province of South Africa. The reasons and the mechanisms underlying the rapid emergence of *C. newmannii* from its indigenous host plants on to sugarcane remain unclear (Javal et al. 2018). Larvae dig galleries into the sugarcane stool, but are, most of the time, found in the below-ground section of the sugarcane stalks. They cause severe crop damage, resulting in ongoing significant economic loss for the growers (Way et al. 2017) and research on biocontrol has shown limited success to date (Javal et al. 2019a). Bio-monitoring of this species is currently done by field surveys, since no lure has yet been found to efficiently monitor adults. Identification of trapped insects can be complicated due to several factors. Firstly, surgarcane agrosystems are usually made of sugarcane fields and natural non-cultivated fields or native forest, which increases the probability to catch a wide variety of species, both from the surrounding natural environment and from the cultivated crops. Secondly, the stage causing the damage is the larva, whose identification, based on morphological features, is very difficult and, therefore, relies heavily on molecular analysis. Finally, the genus *Cacosceles* includes four species that are all distributed in the Afrotropical Region and very difficult to distinguish morphologically by non-specialists, even at the adult stage (Ferreira 1980).

DNA barcoding has been used to accurately identify Cerambycidae pest species (Hodgetts et al. 2016, Wu et al. 2017, Kelnarova et al. 2018), as well as for the recognition of new taxa (Bouyer 2010, Bouyer 2016, Drumont and Ripaille 2019), but the subfamily Prioninae has not received enough attention so far, especially in tropical areas (Jin et al. 2020). The subfamily Prioninae includes more than 1,000 species in about 300 genera (Svacha and Lawrence 2014). They occur mainly in tropical and subtropical areas, but can be found worldwide (Monné et al. 2016). The main aims of this study are: 1) to generate DNA barcodes of the emerging pest *C. newmannii* to facilitate its identification by plant protection officers; 2) to compile a DNA barcoding reference library of other Afrotropical Prioninae to assess the validity of the Barcode Index Number system as a tool to identify them reliably; 3) to assess the level of deep intraspecific lineages that could suggest the existence of cryptic species.

## Material and methods

### Specimen sampling

We built a DNA barcode dataset of 70 specimens of Afrotropical Prioninae, all based on adult specimens mainly collected in South Africa (46), but also from Madagascar (14), Gabon (9) and Republic of the Congo (1).

A total of 21 specimens of *C. newmannii* from South Africa were DNA barcoded: 20 specimens from infested sugarcane fields in Eshowe (KwaZulu-Natal, KZN) and one specimen from the Western Cape. All 21 adult specimens were collected visually during the day, by hand (Suppl. material 1). Thirty-five specimens of other species were collected by attraction to light traps set up using Bioform ‘light towers’ with 15W actinic tubes (https://www.bioform.de) powered with lithium Akku 12V/10.5Ah 116.60Wh batteries (https://www.hellpower.at/) or by free hand.

In addition to the specimens collected in the field, specimens from the dry historical collections, housed in several Natural History Museums or private collections (one specimen deposited at the Durban Natural Science Museum, six specimens at the South African National Collection of Insects, Pretoria, four specimens from Stellenbosch University and three specimens from ND private collection) were also barcoded. Additional details on specimens are given in the dataset DS-AFROPRIO in BOLD.

### Morphological identification

Specimens were identified, based on available literature (Quentin and Villiers 1973, Ferreira 1980), taxonomic expertise of some of the authors (ND) and museum identifications. Most species of Afrotropical Prioninae can be identified, based on examination of external morphological features (concentrated on the head, prothorax and tarsi; Ferreira 1980). In its current concept, the genus *Cacosceles* contains four species divided into two subgenera: *Cacosceles* *sensu stricto* (*C. oedipus* Newman, 1838) and *Zelogenes* Thomson (*C. newmannii* Thomson, 1877; *C. latus* Waterhouse, 1881 and *C. gracilis* Lackerbeck, 2000). The subgenus *Cacosceles sst.* may be easily ditinguished from *Zelogenes*, based on the size and shape of the eyes, the length of antennae and the presence of a tooth on humeral angles (Ferreira 1980). In the subgenus Zelogenes, *C. newmannii* is the only widely distributed and taxonomically well-established species. The two species, *C. latus* and *C. gracilis*, are only known from very few specimens from South Africa and are distinguished from *C. newmannii* by superficial differences in body ratios and punctuation of integument (Ferreira 1980, Lackerbeck 2000). All the *Zelogenes* specimens, examined in the course of this study, were identified as *C. newmannii*. When available, several specimens for each species of Prioninae were sampled from distant locations.

### DNA barcoding

Fresh field specimens were stored in 96% alcohol at 4°C pending DNA extraction. For dry museum material, a hind leg or a tarsus was extracted from specimens and stored dry in an Eppendorf tube. Tissues of 50 specimens were sent to CIRAD (UMR Centre de Biologie pour la Gestion des Populations, Montpellier, France) for DNA barcoding. DNA was extracted non-destructively using a DNeasy Blood & Tissue kit (Qiagen, Hilden, Germany), according to the manufacturer's protocol (with an overnight initial incubation with proteinase K at 56°C / 500 RPM in a ThermoMixer (Eppendorf) and an elution in 100 µl). PCR amplifications were performed using standard primers for barcoding (two parts of mitochondrial cytochrome C oxidase subunit I of invertebrates: LCO1490 and HCO2198 (Folmer et al. 1994) and, when the sequence produced was not clear enough or when DNA was not amplified, Jerry and Pat (Simon et al. 1994). PCR reactions were carried out on a Mastercycler ® Nexus (Eppendorf, Hamburg, Germany) in a volume of 10 μl PCR mix containing 5 μl of Multiplex Master Mix (Qiagen, Hilden, Germany), 0.2 µM of each primers and 1 µl of template DNA. The PCR conditions were as follows: initial DNA denaturation at 94°C for 15 minutes, followed by 35 cycles of 30 s at 94°C, 1 min at annealing temperature (see Table 1) and 1 min at 72°C with a final extension of 20 min at 72°C. The PCR products were sequenced by Eurofins Genomics (http://www.eurofinsgenomics.eu). After extraction, tissues of vouchers specimens were returned to their reference collection of origin.

The remaining 20 samples were shipped to the Canadian Centre for DNA Barcoding (CCDB, Biodiversity Institute of Ontario, University of Guelph) for sequencing. Out of them, 13 samples were sequenced using Single Molecule Real-Time (SMRT) sequencing through the Sequel (PacBio) pipeline at CCDB (Hebert et al. 2018).

### Sequence analysis

Barcode sequences were edited using CodonCode Aligner V.3.7.1. (CodonCode Corporation, Centerville, MA, USA) and checked to identify the presence of pseudogenes using standard detection methods (Haran et al. 2015). Sequences were deposited in BOLD v.4 (www.barcodinglife.com, Ratnasingham and Hebert 2007) and aligned using the 'BOLD aligner' option. Sequences were assigned automatically to Barcode Index Numbers (BINs) in BOLD (Ratnasingham and Hebert 2013). Genetic distances between species were also computed with BOLD using the Kimura 2 Parameter distance model. Neighbour-joining (NJ) trees, used to visualise genetic distances within and between species and sequence clusters, were generated in BOLD. A haplotype network was built using minimum spanning network (epsilon = 0) in PopART (http://popart.otago.ac.nz; Leigh and Bryant 2015).

## Data resources

The resulting sequences, along with the voucher data, images and trace files from Sanger and SMRT sequencing are deposited in the Barcode of Life Database (BOLD, http://www.boldsystems.org) (Ratnasingham and Hebert 2007) and the sequences subsequently deposited in GenBank. All data are available from BOLD at: dx.doi.org/10.5883/DS-AFROPRIO

## Results

We generated a total of 70 COI sequences representing 20 named species (55 sequences identified to species level and remaining sequences identified to genus level) from 16 genera (results on 7 April 2021; Suppl. materials 1, 4). Genetic variability within species ranged from 0 to 14.34% with a mean of 2% (Suppl. material 2). The highest value (14.34%) was observed between two *Mallodon downesi* (Hope, 1843; Suppl. material 3). For *C. newmannii*, genetic distances varied between 0% and 4.8% with a mean of 1.5% (Suppl. material 3).

**Figure 1.**
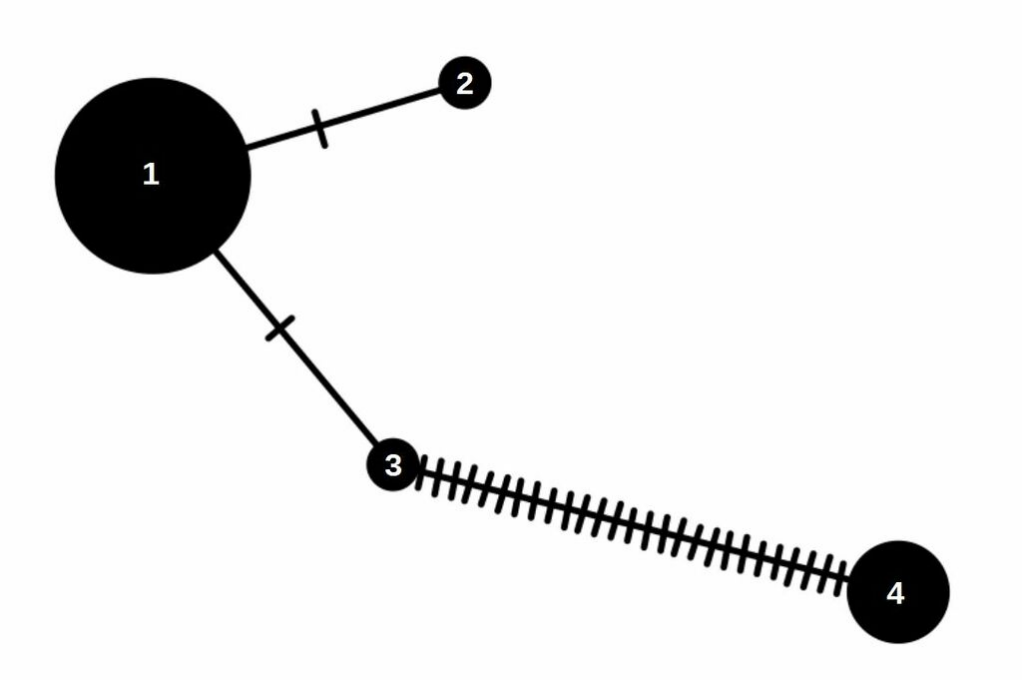
Minimum spanning haplotype network for *C. newmannii* specimens. Mutational steps are symbolised by dashes and the diameter of the circles is proportional to the number of individuals that belong to each haplotype. Specimens belonging to each haplotypes are listed in Table 2.

Sequences were assigned to 42 different BINs, 28 of which were new to BOLD (Suppl. material 4). Out of the 20 named species barcoded, 11 (55%) had their own unique BIN (Suppl. material 5). Seven species (35%) showed deep COI splits with several BINs (Suppl. material 5). The multiple BINs per species ranged from two to a maximum of five within *Mallodon downesi* (Suppl. material 5). *Anomotoma wilwerthi* (Lameere, 1903) showed a "shallow" COI split of only 1.61% between two different BINS (Suppl. materials 5, 6). *Cacosceles newmannii* showed two distinct BINs (BOLD:AEF3555 & BOLD:AEF6074) that diverged between 4.46 and 4.80% (Suppl. materials 5, 6). Out of 21 specimens of *C. newmannii* collected, 20 were sampled in the KZN Province of South Africa and only one in the Western Cape (WCape) Province. Specimens belonging to both BINs have been found sympatrically at Eshowe (South Africa) attacking sugarcane plantations (Suppl. material 1). Four different haplotypes were identifed in *C. newmannii* (Fig. 1, Table 2).

One *Cacosceles* specimen (MJ0009), collected in Eastern Cape, is morphologically similar to *C. oedipus*, but forms its own BIN (BOLD:AEF3200) and shows a high genetic divergence of 12.52% from *C. newmannii* (Suppl. material 5). More specimens are needed to assess its status and it is, therefore, treated here as an unconfirmed candidate species (sensu Lopez-Vaamonde et al. 2021).

## Discussion

DNA barcoding is a major tool in the bio-surveillance of insect pests. It allows rapid identification of an unknown specimen regardless of its developmental stage or state of preservation. DNA barcoding has been extensively used with Cerambycidae for pest diagnostics (Kelnarova et al. 2018) and biosecurity (Hodgetts et al. 2016, Wu et al. 2017). However, reliable molecular identification relies on comprehensive and well-curated databases, that are generally lacking for Prioninae. This study and the resulting barcode database will allow biosecurity officers to easily distinguish any life stages of *C. newmannii* specimens from other common Prioninae species, especially since, based on our data, *C. newmannii* ’s two BINS (BOLD:AEF3555 & BOLD:AEF6074) form a clade.

Interspecific and intraspecific genetic distances observed on this mitochondrial fragment were generally consistent with the currently accepted taxonomy and classification for most species of this subfamily (Ferreira 1980). The genus *Cacosceles* was more intensely sampled. As currently accepted, the genus *Cacosceles* contains four species: *C. newmannii* Thomson, 1877; *C. oedipus* Newman, 1838, *C. latus* Waterhouse, 1881 and *C. gracilis* Lackerbeck, 2000. *C. oedipus* was clearly distinct from *C. newmannii*, based on barcode sequences which confirms their clear species' boundaries. The two species showed 18.35% divergence, based on the barcode fragment. We have sequenced a specimen of a *Cacosceles* from Eastern Cape (MJ0009) that is morphologically similar to *C. oedipus*. This specimen is, however, 20% divergent from the *C. oedipus* sequenced from the Western Cape (MJ0005 & MJ0008). More specimens are needed to assess the status of these divergent BINS. Two *Cacosceles* species, *C. latus* and *C. gracilis*, have not been barcoded yet and a formal taxonomic revision of this genus is needed.

Regarding *C. newmannii*, the distance observed between the two BINS described is above the commonly used threshold of interspecific distance in arthropods (3%; Hebert et al. 2004). Interestingly, the single specimen from the Western Cape Province fell into one of these two clusters. Morphological observations of the specimens, belonging to these clusters, revealed no apparent distinguishing characters. In addition, the specimens of the two clusters from KZN are found in sympatry in infested fields where the species was found feeding on sugarcane. A more detailed study, including more specimens and nuclear genes, is needed to establish if this corresponds to sympatric deep splits and despeciation, lineage fusion events (Hinojosa et al. 2019) or mitochondrial artifacts, such as *Wolbachia* infection.

An extreme case of deep splits within one species is *Mallodon downesii* with five BINs out of six specimens barcoded. *M. downesii* has a very broad distribution across the African continent and is highly polyphagous (Delahaye and Tavakilian 2009). It has also been recorded as an alien introduction to the neotropics, representing a potential danger as a pest of coffee plantations (Wappes et al. 2018). Our barcoding results suggest that it could represent a complex of species that deserves further investigation.

More generally, this study revealed that multiple species of southern African Prioninae show deep intraspecific barcode distances that could not yet be associated with morphological divergences. It should be noted that forests have experienced strong contraction events during past climate oscillations in this region, itself strongly affecting the genetic structure and the speciation of forest insect species (Fabrizi et al. 2019). It is hypothesised that such divergences could originate from a complex evolutionary history of Prioninae, resulting from contraction/extension of their habitat over geological time.

## Conclusions

To complement existing identification tools based on morphology, this barcode database will facilitate clear identification of the most common species of southern African Prioninae, irrespective of taxonomic skills of the observer or developmental stage of the insect. Further analyses using nuclear markers should be carried out to assess the multiple BINs and substantial intraspecific genetic divergences observed within four of the named species of Prioninae found to have multiple BINs. Particular attention should be focused on the pest species *C. newmannii* in order to clarify the species delimitation of the two BINs identified.

## Supplementary Material

1F66BEC1-AA52-543D-A2C4-38A8B3A91833 10.3897/BDJ.9.e64499.suppl1 Supplementary material 1 Metadata of 70 COI sequences and 42 BINs used in this study. Data type Occurrence, taxonomy and DNA sequence data associated with 70 specimens used in our study Brief description Metadata associated with 70 specimens barcoded. All data available via BOLD. File: oo_529525.xlsx https://binary.pensoft.net/file/529525 Javal, M, Terblanche, JS, Conlong, DE, Delahaye, N, Grobbelaar, E, Benoît, L, Lopez-Vaamonde, C, Haran, JM

3003D0B5-B4C2-5ACA-AD0E-067F7F6F192B 10.3897/BDJ.9.e64499.suppl2 Supplementary material 2 Summary of the distribution of sequence divergence (Kimura 2 Parameter) at each taxonomic level (species, genus and famlily) Data type Genetic divergence Brief description The Distance Summary reports the sequence divergence between barcode sequences at the species, genus and family level and also contrasts the distribution of within-species divergence to between-species divergence. File: oo_509980.pdf https://binary.pensoft.net/file/509980 Javal, M, Terblanche, JS, Conlong, DE, Delahaye, N, Grobbelaar, E, Benoît, L, Lopez-Vaamonde, C, Haran, JM

7AE03136-D752-558E-8844-5E3CE94A85AB 10.3897/BDJ.9.e64499.suppl3 Supplementary material 3 Detailed genetic distance table at species level Data type K2P genetic distances Brief description genetic distances (K2P) calculated by BOLD for 234 comparisons amongst 42 sequences File: oo_509899.xls https://binary.pensoft.net/file/509899 Javal, M, Terblanche, JS, Conlong, DE, Delahaye, N, Grobbelaar, E, Benoît, L, Lopez-Vaamonde, C, Haran, JM

FEA22597-AF35-565B-B4F2-38FC64CCEC0D 10.3897/BDJ.9.e64499.suppl4 Supplementary material 4 Details about 42 Barcode Index Numbers (BINs) analysed in this study Data type Number of COI sequences per BIN Brief description Number of COI sequences for 28 BINs unique to our project and therefore new to BOLD and 14 BINs already present in BOLD File: oo_529526.xls https://binary.pensoft.net/file/529526 Javal, M, Terblanche, JS, Conlong, DE, Delahaye, N, Grobbelaar, E, Benoît, L, Lopez-Vaamonde, C, Haran, JM

386B7C7F-8966-56CB-97DE-78FA55BCBF84 10.3897/BDJ.9.e64499.suppl5 Supplementary material 5 Summary statistics for specimens included in this study. BIN Average: average intraspecific distance within BIN; BIN Dmax = maxim intraspecific distance within a BIN; Dmin_NN heterospecific = minimum distance to nearest neighbour; NN= nearest neighbour. Data type summary on genetic data Brief description A summary on the number of specimens per BIN, species that are non-monophyletic, which species have their own unique BIN and geographic distribution of each BIN. File: oo_529610.xlsx https://binary.pensoft.net/file/529610 Javal, M, Terblanche, JS, Conlong, DE, Delahaye, N, Grobbelaar, E, Benoît, L, Lopez-Vaamonde, C, Haran, JM

25F3C554-3411-5E27-AF01-2A9AF6E856F6 10.3897/BDJ.9.e64499.suppl6 Supplementary material 6 Neighbour-Joining phylogram of the 70 sequences analysed (Distance Model : Kimura 2 Parameter). Data type Phylogenetic tree reconstructed using COI sequences Brief description Neighbour-Joining tree reconstructed in BOLD, using 70 COI sequences File: oo_529609.pdf https://binary.pensoft.net/file/529609 Javal, M, Terblanche, JS, Conlong, DE, Delahaye, N, Grobbelaar, E, Benoît, L, Lopez-Vaamonde, C, Haran, JM

## Figures and Tables

**Table 1.** PCR primers and conditions.

Primer		Annealing temperature	Reference
HCO2198	**CAGGAAACAGCTATGAC**TAAACYTCDGGATGBCCAAARAATCA **CAGGAAACAGCTATGAC**TAAACYTCAGGATGACCAAAAAAYCA **CAGGAAACAGCTATGAC**TAAACTTCWGGRTGWCCAAARAATCA	52°C	Folmer et al. 1994 modified in Germain et al. 2013
LCO1490	**TGTAAAACGACGGCCAGT**TTTCAACTAAYCATAARGATATYGG **TGTAAAACGACGGCCAGT**TTTCAACWAATCATAAAGATATTGG		
Jerry	CAACATTTATTTTGATTTTTTGG	55°C	Simon et al. 1994
Pat	TCCAATGCACTAATCTGCCATATTA		

M13 tails from Ivanova et al. (2007) are in bold.

**Table 2.** Collection data and genetic information regarding *Cacosceles newmannii* samples. Haplotypes numbers refer to Fig. 1.

**Sample ID**	**BIN**	**Haplotype**	**Collection Date**	**Country**	**State/Province**	**Sector**
MJ0002	BOLD:AEF3555	1	2018	South Africa	KwaZulu Natal	Entumeni
MJ0003	BOLD:AEF6074	4	2018	South Africa	KwaZulu Natal	Entumeni
MJ0004	BOLD:AEF6074	4	2018	South Africa	KwaZulu Natal	Entumeni
MJ0006	BOLD:AEF3555	1	25-Feb-2018	South Africa	Western Cape	Stellenbosch
MJ0007	BOLD:AEF3555	1	17-Feb-2017	South Africa	KwaZulu Natal	Entumeni
MJ0010	BOLD:AEF3555	1	17-Feb-2017	South Africa	KwaZulu Natal	Entumeni
MJ0038	BOLD:AEF6074	4	2018	South Africa	KwaZulu Natal	Entumeni
MJ0039	BOLD:AEF3555	1	17-Feb-2017	South Africa	KwaZulu Natal	Entumeni
MJ0040	BOLD:AEF3555	1	17-Feb-2017	South Africa	KwaZulu Natal	Entumeni
MJ0041	BOLD:AEF3555	1	17-Feb-2017	South Africa	KwaZulu Natal	Entumeni
MJ0042	BOLD:AEF3555	2	17-Feb-2017	South Africa	KwaZulu Natal	Entumeni
MJ0043	BOLD:AEF3555	1	17-Feb-2017	South Africa	KwaZulu Natal	Entumeni
MJ0044	BOLD:AEF3555	3	17-Feb-2017	South Africa	KwaZulu Natal	Entumeni
MJ0045	BOLD:AEF3555	1	17-Feb-2017	South Africa	KwaZulu Natal	Entumeni
MJ0046	BOLD:AEF6074	4	2018	South Africa	KwaZulu Natal	Entumeni
MJ0047	BOLD:AEF3555	1	2018	South Africa	KwaZulu Natal	Entumeni
MJ0048	BOLD:AEF3555	1	2018	South Africa	KwaZulu Natal	Entumeni
MJ0049	BOLD:AEF3555	1	2018	South Africa	KwaZulu Natal	Entumeni
MJ0051	BOLD:AEF3555	1	2018	South Africa	KwaZulu Natal	Entumeni
MJ0052	BOLD:AEF3555	1	2018	South Africa	KwaZulu Natal	Entumeni
MJ0053	BOLD:AEF3555	1	2018	South Africa	KwaZulu Natal	Entumeni
