# Supplementary material for: DNA barcoding for bio-surveillance of emerging pests and species identification in Afrotropical Prioninae (Coleoptera, Cerambycidae)
Source: Biodivers Data J. 2021 Apr 28;9:e64499. doi: 10.3897/BDJ.9.e64499 (PMC8099841; doi:10.3897/BDJ.9.e64499)
Supplement: Supplementary material 2 — Summary of the distribution of sequence divergence (Kimura 2 Parameter) at each taxonomic level (species, genus and famlily) [file bdj-09-e64499-s002.pdf]

Distance Summary Result

DS-AFROPRIO (70 sequences)

Distance Summary Tables

The distribution of sequence divergence at each taxonomic level is summarized below. Detailed distance tables can be downloaded by clicking on Details button for each rank.

Label	n	Taxa	Comparisons	Min Dist(%)	Mean Dist(%)	Max Dist(%)	SE Dist(%)
Within Species	42	9	234	0.00	2.02	14.34	0.01
Within Genus	31	3	52	14.57	19.82	21.19	0.03
Within Family	53	1	1092	16.68	26.41	32.35	0.00

Distance Summary Graphs

Sequence divergence for all sequences compared at the species and genus levels.

Within Species

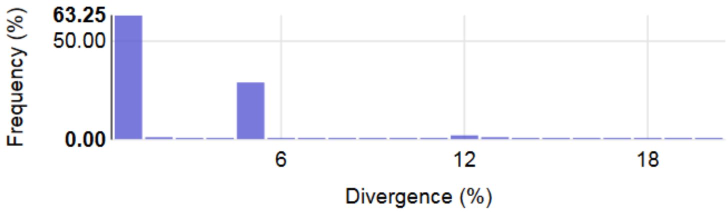

Within Genus

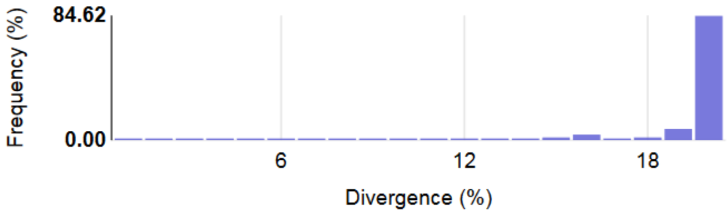

Normalized Results

The within-species distribution is normalized to reduce bias in sampling at the species level. The table below summarizes this distribution, while the histogram plots the distribution of normalized divergence for species (blue) against the genus divergences (red).

Normalized Divergence Statistics

Quantity	Value
Species Count	9
Mean Within-Species Dist (%)	2.83
SE of Mean Within-Species Dist (%)	0.45
Min Between-species Dist (%)	14.57

Normalized Divergence Histogram

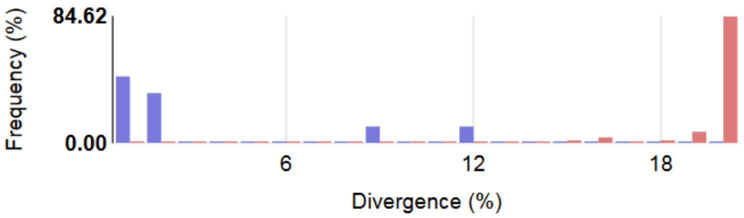

Analysis description

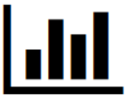

The Distance Summary reports the sequence divergence between barcode sequences at the species, genus and family level, and also contrasts the distribution of within-species divergence to between-species divergence.

Distance Model: Kimura 2 Parameter
Marker: COI-5P
Deletion Method: Pairwise Deletion
Minimum Complete Columns: 0
Group By: Geography: GPS
Alignment: BOLD Aligner (Amino Acid based HMM)
Filters Applied: ≥ 200bp only
