# Supplementary material for: DNA barcoding for bio-surveillance of emerging pests and species identification in Afrotropical Prioninae (Coleoptera, Cerambycidae)
Source: Biodivers Data J. 2021 Apr 28;9:e64499. doi: 10.3897/BDJ.9.e64499 (PMC8099841; doi:10.3897/BDJ.9.e64499)
Supplement: Supplementary material 6 — Neighbour-Joining phylogram of the 70 sequences analysed (Distance Model : Kimura 2 Parameter). [file bdj-09-e64499-s006.pdf]

BOLD TaxonID Tree

Title	:	Tree Result - DS-AFROPRIO (70 records selected)
Date	:	07-Apr-2021
Data Type	:	Nucleotide
Distance Model	:	Kimura 2 Parameter
Marker	:	COI-5P
Colourization	:	[blue]=Stop Codons [red]=Contamination or misidentification

Label	:	Sample ID
Label	:	Taxon
Label	:	Country
Label	:	Barcode Cluster (BIN)

Sequence Count	:	70
Species count	:	20
Genus count	:	16
Family count	:	1
Unidentified	:	17

BIN Count	:	42

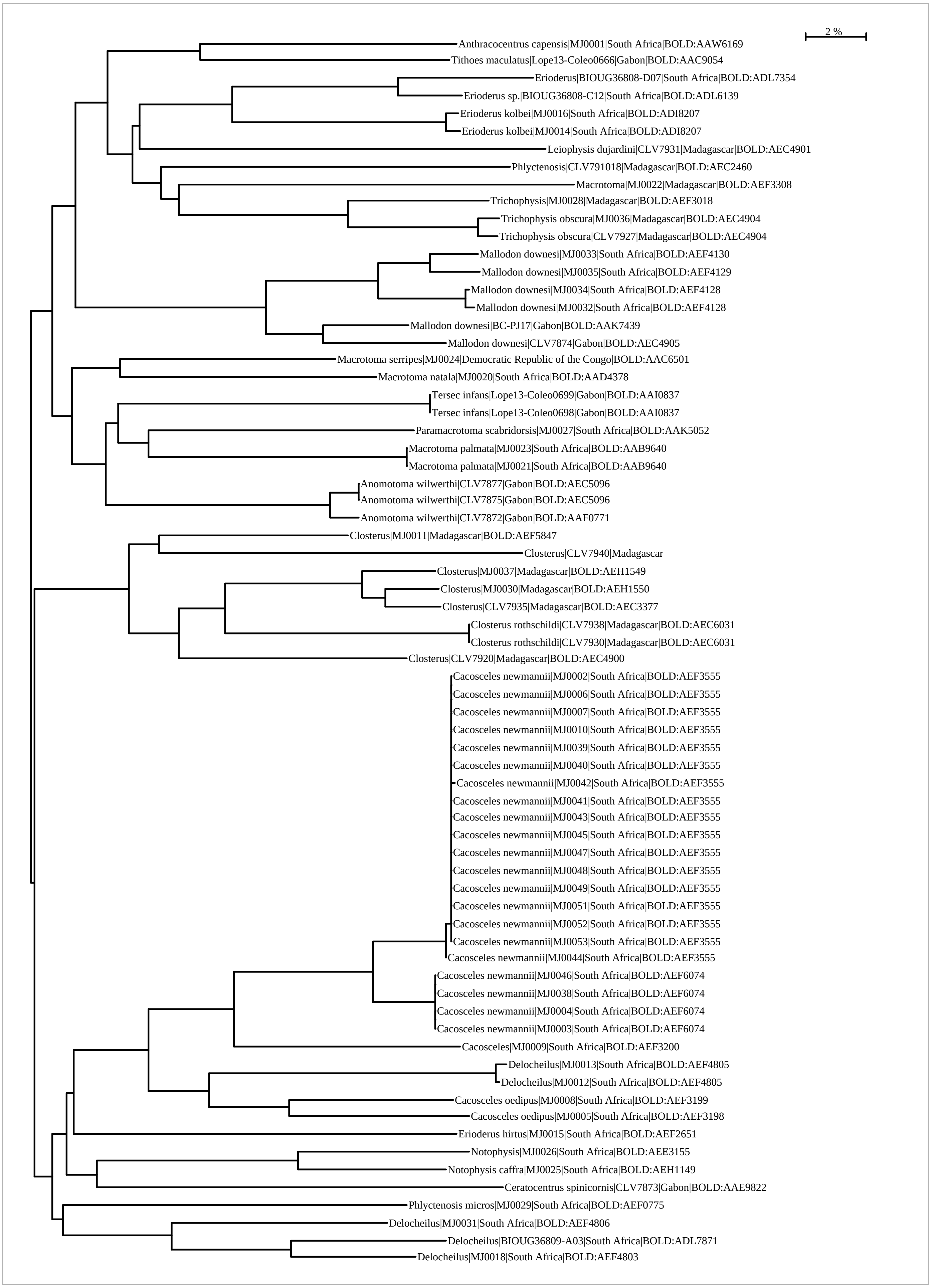
2 %
Anthracocentrus capensis|MJ0001|South Africa|BOLD:AAW6169
Tithoes maculatus|Lope13-Coleo0666|Gabon|BOLD:AAC9054
Erioderus|BIOUG36808-D07|South Africa|BOLD:ADL7354
Erioderus sp.|BIOUG36808-C12|South Africa|BOLD:ADL6139
Erioderus kolbei|MJ0016|South Africa|BOLD:ADI8207
Erioderus kolbei|MJ0014|South Africa|BOLD:ADI8207
Leiophysis dujardini|CLV7931|Madagascar|BOLD:AEC4901
Phlyctenosis|CLV791018|Madagascar|BOLD:AEC2460
Macrotoma|MJ0022|Madagascar|BOLD:AEF3308
Trichophysis|MJ0028|Madagascar|BOLD:AEF3018
Trichophysis obscura|MJ0036|Madagascar|BOLD:AEC4904
Trichophysis obscura|CLV7927|Madagascar|BOLD:AEC4904
Mallodon downesi|MJ0033|South Africa|BOLD:AEF4130
Mallodon downesi|MJ0035|South Africa|BOLD:AEF4129
Mallodon downesi|MJ0034|South Africa|BOLD:AEF4128
Mallodon downesi|MJ0032|South Africa|BOLD:AEF4128
Mallodon downesi|BC-PJ17|Gabon|BOLD:AAK7439
Mallodon downesi|CLV7874|Gabon|BOLD:AEC4905
Macrotoma serripes|MJ0024|Democratic Republic of the Congo|BOLD:AAC6501
Macrotoma natala|MJ0020|South Africa|BOLD:AAD4378
Tersec infans|Lope13-Coleo0699|Gabon|BOLD:AAI0837
Tersec infans|Lope13-Coleo0698|Gabon|BOLD:AAI0837
Paramacrotoma scabridorsis|MJ0027|South Africa|BOLD:AAK5052
Macrotoma palmata|MJ0023|South Africa|BOLD:AAB9640
Macrotoma palmata|MJ0021|South Africa|BOLD:AAB9640
Anomotoma wilwerthi|CLV7877|Gabon|BOLD:AEC5096
Anomotoma wilwerthi|CLV7875|Gabon|BOLD:AEC5096
Anomotoma wilwerthi|CLV7872|Gabon|BOLD:AAF0771
Closterus|MJ0011|Madagascar|BOLD:AEF5847
Closterus|CLV7940|Madagascar
Closterus|MJ0037|Madagascar|BOLD:AEH1549
Closterus|MJ0030|Madagascar|BOLD:AEH1550
Closterus|CLV7935|Madagascar|BOLD:AEC3377
Closterus rothschildi|CLV7938|Madagascar|BOLD:AEC6031
Closterus rothschildi|CLV7930|Madagascar|BOLD:AEC6031
Closterus|CLV7920|Madagascar|BOLD:AEC4900
Cacosceles newmannii|MJ0002|South Africa|BOLD:AEF3555
Cacosceles newmannii|MJ0006|South Africa|BOLD:AEF3555
Cacosceles newmannii|MJ0007|South Africa|BOLD:AEF3555
Cacosceles newmannii|MJ0010|South Africa|BOLD:AEF3555
Cacosceles newmannii|MJ0039|South Africa|BOLD:AEF3555
Cacosceles newmannii|MJ0040|South Africa|BOLD:AEF3555
Cacosceles newmannii|MJ0042|South Africa|BOLD:AEF3555
Cacosceles newmannii|MJ0041|South Africa|BOLD:AEF3555
Cacosceles newmannii|MJ0043|South Africa|BOLD:AEF3555
Cacosceles newmannii|MJ0045|South Africa|BOLD:AEF3555
Cacosceles newmannii|MJ0047|South Africa|BOLD:AEF3555
Cacosceles newmannii|MJ0048|South Africa|BOLD:AEF3555
Cacosceles newmannii|MJ0049|South Africa|BOLD:AEF3555
Cacosceles newmannii|MJ0051|South Africa|BOLD:AEF3555
Cacosceles newmannii|MJ0052|South Africa|BOLD:AEF3555
Cacosceles newmannii|MJ0053|South Africa|BOLD:AEF3555
Cacosceles newmannii|MJ0044|South Africa|BOLD:AEF3555
Cacosceles newmannii|MJ0046|South Africa|BOLD:AEF6074
Cacosceles newmannii|MJ0038|South Africa|BOLD:AEF6074
Cacosceles newmannii|MJ0004|South Africa|BOLD:AEF6074
Cacosceles newmannii|MJ0003|South Africa|BOLD:AEF6074
Cacosceles|MJ0009|South Africa|BOLD:AEF3200
Delocheilus|MJ0013|South Africa|BOLD:AEF4805
Delocheilus|MJ0012|South Africa|BOLD:AEF4805
Cacosceles oedipus|MJ0008|South Africa|BOLD:AEF3199
Cacosceles oedipus|MJ0005|South Africa|BOLD:AEF3198
Erioderus hirtus|MJ0015|South Africa|BOLD:AEF2651
Notophysis|MJ0026|South Africa|BOLD:AEE3155
Notophysis caffra|MJ0025|South Africa|BOLD:AEH1149
Ceratocentrus spinicornis|CLV7873|Gabon|BOLD:AAE9822
Phlyctenosis micros|MJ0029|South Africa|BOLD:AEF0775
Delocheilus|MJ0031|South Africa|BOLD:AEF4806
Delocheilus|BIOUG36809-A03|South Africa|BOLD:ADL7871
Delocheilus|MJ0018|South Africa|BOLD:AEF4803
